# Supplementary material for: Leishmania braziliensis causing human disease in Northeast Brazil presents loci with genotypes in long-term equilibrium
Source: PLoS Negl Trop Dis. 2022 Jun 15;16(6):e0010390. doi: 10.1371/journal.pntd.0010390 (PMC9239440; doi:10.1371/journal.pntd.0010390)
Supplement: Attachment — Submitted filename: Responses to reviewers_PNTD-D-21-00503.docx [file pntd.0010390.s001.docx]

PNTD-D-21-00503

We would like to deeply thank both reviewers for their careful consideration of the manuscript (ms) and for their very constructive comments. We made the effort to incorporate every suggestion made in the currently revised version of the ms. Below, we respond in a point-by-point fashion to each reviewer and indicate where the modifications can be found in the revised ms. The modifications are highlighted in yellow in the ms. After the responses to the two reviewers, a third section can be found below, in which we briefly describe four minor corrections that we also made to the original submission.

REVIEWER 1

GENERAL COMMENTS.

The paper was well written and the text is very clear, allowing publication without revision of the english language. The objectives of the study is clearly articulated with a clear testable answers and the study design is appropriate to address the stated objectives. The study population is especially important because it is a community village in the northeast region of Brazil, an extremely vulnerable population and an important endemic area of ATL. The study population is clearly described and appropriate for the hypothesis being tested. I emphasize that the sample size used in this work is very substantial and sufficient to ensure adequate power to address the hypothesis being tested, which further reinforces the quality of the work. However I think that the loci selected for the study might be too small region/sequence to investigate if there is sex-like recombination in a complex population of Leishmania. This would be an important bias of the work. The authors need to comment further on how the molecular targets were selected for this work. And also discuss this possible bias. The statistical analysis were correctly used to support conclusions and all ethical requirements have been met. Moreover, presented a methodology suitable for evaluating these purposed aims; the results allowed a very detailed argumentation of the data in a holistic way.

RESPONSE: We deeply thank the reviewer for the detailed evaluation of the manuscript and for the constructive comments.

The markers used in the current research derive from two previous studies in which we began to characterize the *L. braziliensis* population endemic in the study region at Northeast Brazil, listed as references 5 and 15 in the submitted manuscript. In summary, we randomly amplified, cloned, sequenced and compared several regions of the parasites’ genomes. Then, we searched for haplotypes of single nucleotide polymorphisms / indels that could reproducibly discriminate strains among individuals of that population. We detected six loci with such haplotypes that distributed over five different chromosomes of *L. braziliensis* (reference 5).

In the current research, we opted to use markers of that panel that fell within previously annotated genes located in two different chromosomes of the parasite. They marked the putative coding regions annotated for Pyruvate Dehydrogenase Kinase (CHR24/3074 haplotype) and for Long-chain Fatty Acidcoenzyme A Ligase (CHR28/425451 haplotype). In this way, we attempted to partly mitigate potentially negative consequences of using anonymous markers, like the ones that fell on chromosomes 26, 32 and 35 in our previous report (reference 5). One such consequence we tried to overcome was a high mutation rate that would cause the continuous emergence of new haplotypes in the population if the marker happened to fall on a non-coding region of the parasite’s genome.

The current study does not attempt to give a definitive answer as to the modes of reproduction of *L. braziliensis*. We believe a genome-wide approach as necessary to appropriately address this issue, given particularities of Trypanosomatids like the organization and expression of their genomes. In the current status of such knowledge, though, we think this research helps reinforce the amounting evidence that some sort of sex-like reproduction seems to occur at some point of the parasite’s life cycle.

However, our ultimate focus is on the practical consequences that frequent genetic exchange may present for the management of the endemics, particularly regarding predictive diagnosis. Patients of ATL may evolve to severe forms of disease and present refractoriness to some of the drugs used to treat them. So, the development of diagnostic protocols capable of predicting these outcomes is most welcome in the field. Although we do not envision CHR24/3074 and CHR28/425451 use for diagnosis, our findings that some chromosomal markers may be in Hardy Weinberg Equilibrium in a population of *L. braziliensis* highlight the utmost importance of thoroughly testing the short- and long-term accuracy of predictive diagnostic tools based on nucleic acid detection before their roll out to the ATL affected foci.

All in all, we agree that the concerns raised by the reviewer are sound and sought to ameliorate them by adding slightly modified versions of the four paragraphs above to the revised manuscript. The first two paragraphs above appear in sequence within the topic ‘Genotyping *L. braziliensis* isolates’ of Methods, between lines 190 and 206 of the revised ms. The two last paragraphs appear in Discussion, between lines 535 and 551 of the revised ms.

SPECIFIC COMMENTS.

1. Lines 114-117: I think this might be too small a region/sequence to investigate if there is sex-like recombination in a complex population of Leishmania. This would be an important bias of the work. In the methodology, the authors need to comment further on how the molecular targets were selected for this work. And also discuss this possible bias.

RESPONSE: We thank the reviewer for the suggestion. We have partially addressed it when discussing about the general comment above. Besides the new paragraphs that address these issues mentioned in our response to the general comment, which appear in the topic ‘Genotyping L. braziliensis isolates’ of Methods between lines 190 and 206 and in Discussion between lines 535 and 551 of the revised ms, we added a paragraph to Discussion between lines 524 and 534, in which we emphasize that even other loci that we had previously described might have presented different results from those of CHR24/3074 and CHR28/425451, but also explain the reasons for compromising and opting for these two loci in our approach.

2. Lines 123-130: Please add the related reference to the information presented about the study area.

RESPONSE: We have added the citations to references 16 and 17, already listed in the original ms, to the end of the ‘Study area’ section in the revised ms. Those two reports explain in detail the geography and dynamic behavior of ATL in the affected region. Those references are:

- (16) Schriefer A, Guimaraes LH, Machado PR, Lessa M, Lessa HA, Lago E, et al. Geographic clustering of leishmaniasis in northeastern Brazil. Emerg Infect Dis. 2009/06/16. 2009;15: 871–876. doi:10.3201/eid/1506.080406.
- (17) Silva J, Queiroz A, Moura I, Sousa RS, Guimarães LH, Machado PRL, et al. Dynamics of American tegumentary leishmaniasis in a highly endemic region for Leishmania (Viannia) braziliensis infection in northeast Brazil. PLoS Negl Trop Dis. 2017. doi:10.1371/journal.pntd.0006015

3. Lines 326-328: I suggest that the authors explain here which values would indicate the existence of LD and which values are assumed to be absence of LD.

RESPONSE: We agree with the reviewer that disclosing the values associated with LD will improve readers understanding of that fragment in the main text. We edited that fragment in the revised ms to read ‘Further suggesting that genetic recombination within that natural population of L. braziliensis is frequent, CHR24/3074 polymorphisms did not show linkage disequilibrium with those of CHR28/42451, as indicated by pair-wise comparison values above 0.05 in table 2. As expected, sets of polymorphic nucleotides within each locus were in LD, displaying pair-wise comparison values below 0.05 in table 2, in accordance to our option of using haplotypes defined by biallelic positions at each locus instead of just alleles in individual polymorphic positions in our population analyses.’ This fragment can be found between lines 348 and 355 of the revised ms. The sentences containing the reviewer’s suggestion are highlighted above.

4. Lines 397-399: I suggest adding more recent articles on this topic: Molecular tools confirm natural Leishmania (Viannia) guyanensis/L. (V.) shawi hybrids causing cutaneous leishmaniasis in the Amazon region of Brazil (Lima et. al., 2021); Phenotypic characterization of Leishmania spp. causing cutaneous leishmaniasis in the lower Amazon region, western Pará state, Brazil, reveals a putative hybrid parasite, Leishmania (Viannia) guyanensis × Leishmania (Viannia) shawi shawi (Jennings et. al., 2014).

RESPONSE: We promptly included these very well reminded reports to the references list. They consist in references 31 and 32 of the revised ms.

5. Lines 404-410: One of the most important assumptions of the Hardy-Weinberg equilibrium is the absence of gene flow. This disagrees with his statement that sexual and asexual reproduction may be equally relevant to the life cycle of the parasites and that the findings for parasites isolated from patients in this study support this hypothesis. Since it is not possible for a population to be in HWE and at the same time have sexual reproduction, I ask the authors to clarify better this statement further.

RESPONSE: We thank the reviewer for the comment and realize that we were not conveying a clear message to the reader at that point in Discussion. So, to improve clarity we adjusted that paragraph to read as below. The modified fragment is underlined in this excerpt and consists in lines 427 to 432 of the revised ms.

‘Recent experimental research has shaken the dogma showing that *L. major* and possibly other species of Leishmania are indeed capable of mating and leading to hybrid offspring within sand flies [11,12]. These reports suggest that both sexual and asexual reproduction may be relevant to the life cycle of the parasites albeit occurring within distinct hosts in natural populations of the protozoa. Good examples of human disease-causing protozoa that share this reproduction pattern are the *Plasmodium* spp. They multiply asexually by schizogony within human hosts and sexually during sporogony in the invertebrate hosts.’

6. Lines 654-664 / Figure 2: In my opinion this figure is not the best way to demonstrate the nucleotide haplotype data found in the two chromosome loci studied. I suggest using the cluster dendrogram presentation, or that the authors propose another possibility to show these results.

RESPONSE: We changed the phylogenetic tree of the haplotypes in Figure 2 from circular to traditional (i.e., squared), as suggested by the reviewer.

----//----//----

REVIEWER 2

COMMENTS

1. How do the results compare with previously published studies on *L braziliensis* that examined microsatellite loci to investigate linkage disequilibrium in Peru and Bolivia [Rougeron et al, PNAS 2009]? How are the ~600nt genetic loci on chromosomes 24 and 28 likely to reveal new insights?

RESPONSE: We thank the reviewer for indicating the need to confront the findings between the pioneering work of Rougeron et al. and the current report. We attempted that in the three paragraphs below, which now appear in tandem between lines 444 and 479 of the revised ms.

‘A pioneering study had previously assessed the population genetics of L. braziliensis, exploring a large panel of micro-satellite markers and F statistics [36]. The research included individuals from four populations located in the Amazon, sampled from one focus of ATL in Peru and one in Bolivia, spanning about 100 to 200 Km2 each and yielding 124 parasite isolates. Its major conclusions were that sex-like reproduction with intense inbreeding among strains of the parasite occurred in those populations. The authors also found a suppression in heterozygous genotypes in comparison with what would be expected by Hardy-Weinberg prediction, and evidence of strong structralization reflected in the observation of multiple clusters of individuals in the samples analyzed. They inferred that these clusters might be consequences of the existence of several different ecotypes in the area of sampling within the highly diversified flora and fauna of the rain forest.

In the current study, we revisited the population genetics of L. braziliensis in a rural setting hyperendemic for ATL that spans for 10,000 Km2, close to the Atlantic Ocean in Northeast Brazil. We employed two sets of haplotypes of six and three SNPs or indels that marked stretches of precisely 573 and 515 nucleotides (i.e., measured from first to last SNP / indel in the locus) in chromosomes 24 and 28 of the parasites, respectively. Overall, we detected that these two loci were in HWE, reinforcing the conclusion on the existence of sex-like reproduction in part of the L. braziliensis life-cycle made by Rougeron et al.[36]. However, we did not find evidence of heterozygous suppression or structuralization in that population.

We believe that the partial discrepancy in these observations is not related to the different molecular and statistical approaches of the two studies. We think that the major driver for the partially contrasting observions is the difference between the highly diversified ecosystem of the Amazon rain forest represented in Rougeron et al.[36] and the much less heterogeneous fauna and flora of a rural area dominated by farms with residual forest coverage and small to mid-sized villages in the current study. It is also likely that human beings dwelling in this ATL hyperendemic region acquire the parasites both while working within the residual forests, used to grow cocoa crops, as well as in the vicinities of their homes, what coupled to the intense day-to-day movement of these individuals within and between villages of the region may cause the more even distributions and prevent the clustering of genotypes, as we detected in the current study. All in all, we believe that the two studies offer insights of complementary realms that coexist in the transmission cycles of Leishmania spp.’

2. Is the lack of linkage disequilibrium between the parasite samples due to the targets selected for investigation? Could inclusion of other potential targets as described in the previous studies by the authors changed the outcome?

RESPONSE: The reviewer’s comment is very appropriate. We cannot rule out that the use of other markers we had previously detected in other chromosomes of the parasite [reference 5 in the ms] might have led to different outcomes, namely that part or all of them would not present Hardy Weinberg Equilibrium and present linkage disequilibrium in the study *L. braziliensis* population. It is important to point out that the approach we opted for was very conservative, consisting in culturing over 150 isolates of *L. braziliensis*, then cloning over 300 inserts into plasmid vectors and finally sequencing over 1,800 clones. So, it ended up neither practical nor cost effective for us to expand the analyses to the other four loci we had previously identified [5]. We compromised opting for two loci that located in two different chromosomes and were both marking putative coding regions previously annotated in the *L. braziliensis* genome. For complete disclosure to the readers, we added this explanation to Discussion in the revised manuscript. It consists in the paragraph located between lines 524 and 534 of the revised ms.

Nevertheless, eventual findings observable with the other four loci that would potentially deviate from what was detected for CHR24/3074 and CHR28/425451 would not change the ultimate message in the manuscript that at least some regions in the *L. braziliensis* genome should be expected to be in HWE in parasite populations found in affected foci. As a result, it demands that any diagnostic test developed for prognosticating outcomes of human infection with these parasites should be carefully assessed for long-term accuracy. This has been emphasized in the two final paragraphs of the revised manuscript, found between lines 542 and 568 of the revised paper. The paragraph between lines 542 and 551 was added to the revised ms, while that between lines 552 and 568 was already present in the original submission.

3. Are the temporally distinct sample sets collected almost a decade apart have gone through a number of in vitro replication cycles. How did the authors ensure the validity of the results? Was it done by random assignment of the testing set and validation sets of samples?

RESPONSE: The concern raised by the reviewer regarding the *L. braziliensis* isolates is also appropriate. All DNA used in the study was extracted from freshly isolated parasites in the primary sample, collected between 2008 and 2011, and from first thaw from first passaged -180C frozen isolates in the validation sample, collected between 1999 and 2001. This strategy aimed to avoid the accumulation of polymorphisms in the genomes of isolates undergoing multiple culture passages, and thus to keep them best reflecting their natural genotypes in the affected focus. We clarified this point in the ‘Parasites’ section of Methods in the revised manuscript, between lines 166 and 179, which now reads:

‘The L. braziliensis isolates used in the study were cultured from aspirates of the borders of skin and mucosal lesions. Aspirated material was immediately suspended in biphasic liver infusion tryptose/Novy, McNeal, Nicolle (LIT/NNN) medium and incubated at 26°C for two to three weeks. The suspension was then transferred to complete Schneider’s medium supplemented with 10% heat-inactivated fetal calf serum and Gentamicin 50 mg/mL (Sigma-Aldrich) and incubated at 26°C for up to another two weeks. Parasite stocks were frozen without further subculture in 10% DMSO / 90% growth medium in liquid nitrogen. All DNA used in the study was extracted from freshly isolated parasites in the primary sample, collected between 2008 and 2011, and from first thaw from first passaged frozen isolates in the validation sample, collected between 1999 and 2001. This strategy aimed to avoid the accumulation of polymorphisms in the genomes of isolates undergoing multiple culture passages. The isolates DNA were employed for species and genotype determination as described below.’

Please, note that the underlined fragment above was added to the original paragraph (not underlined) in the revised ms.

Regarding the validation of findings, we performed it by carrying out the population genetics analyses in parallel in two separate samples of *L. braziliensis*. The primary sample consisted in 120 isolates collected between 2008 and 2011. The validation sample consisted in 35 isolates collected between 1999 and 2001. We didn’t find discrepancies in the observations between samples.

4. Are there any current diagnostics assays based on the genetic loci from chromosomes 24 and 28 or the authors are making a more generalized statement with respect to the diversity of the loci studied. The authors need to make a clear case for the utility of the findings of the study.

RESPONSE: We attempted to emphasize the translational relevance of our findings in the last paragraph of the original manuscript. We thank the reviewer’s observation and, since we think it will probably be shared by readers, we attempted to further convey the message of a translational goal by adding the text below, which immediately precedes and complements the content in the final paragraph of the revised paper. This paragraph can be found between lines 542 and 551 of the revised ms.

‘Our ultimate focus is on the practical consequences that frequent genetic exchange may present for the management of the endemics. Particularly regarding predictive diagnosis. Patients of ATL may evolve to severe forms of disease and present refractoriness to some of the drugs used to treat them. So, the development of diagnostic protocols capable of predicting these outcomes is most welcome in the field. Although we do not envision CHR24/3074 and CHR28/425451 use for diagnosis, our findings that some chromosomal markers may be in Hardy Weinberg Equilibrium in a population of *L. braziliensis* highlight the utmost importance of thoroughly testing the short- and long-term accuracy of predictive diagnostic tools based on nucleic acid detection, before their roll out to the ATL affected foci.’

----//----//----

AUTHORS EDITS TO THE ORIGINAL SUBMISSION

While revising the ms to incorporate the reviewers’ suggestions we also made the four minor modifications listed below to the revised ms.

1) In the sentence that appears in lines 164 and 165 of the revised ms we substituted the word ‘secondary’ to ‘validation’ to refer to the sample of parasites collected between 1999 and 2001. This modification was to maintain conformity with the rest of the text.

2) In table 1 of the original submission, we had left by mistake the observed and expected counts of genotypes of a preliminary evaluation of CHR28/425451 that employed only 85 isolates of *L. braziliensis*. In those fields we could find: N=85, observed counts of 25, 49 and 11, expected counts of 29, 41 and 15 for CCT:CCT, CCT:TT-, TT-:TT-, respectively. In the revised ms these fields now consist of: N=85, observed counts of 34, 68 and 15, expected counts of 40, 57, 21 for CCT:CCT, CCT:TT-, TT-:TT-, respectively. The HWE p values did not change when the entire dataset of 117 isolates was used (HWE p=0.30; HWE/MCMC p=0.06).

3) The update of the dataset for CHR28/425451 from 85 to 117 isolates also slightly affected the results obtained for Heterozygosity and Fst, which in the original submission consisted of HT=0.4965, HS=0.4885 and Fst=0.02, while in the revised ms they appear as HT=0.4951, HS=0.4883 and Fst=0.01 at lines 388 and 389.

4) In table 2, the column and line corresponding to the polymorphic position 604 in CHR24/3074 were missing in the original submission. They were added to the table 2 of the revised ms. This omission affected only the presentation of the table, not affecting the actual linkage disequilibrium analyses that took into account all six polymorphic positions in CHR24/3074 and all three in CHR28/425451.
